# Giant lipoma in the deep palmar compartment: surgical challenges

**DOI:** 10.1093/jscr/rjaf849

**Published:** 2025-10-28

**Authors:** Ritwik Kaushik, Ankush Nayyar, Haradanahalli Nagaraju Pavithra

**Affiliations:** Department of Plastic and Reconstructive Surgery, Indus International Hospital, Chandigarh - Ambala Road, National Highway No. 22, Derabassi, 140507, Punjab, India; Department of Research and Publications, Indus Hospitals, Phase 6, Sector 56, Sahibzada Ajit Singh Nagar, 160055, Punjab, India; Department of Pathology, Indus Super Specialty Hospital, opp. Old DC Office, Phase 1, Sahibzada Ajit Singh Nagar, 160055, Punjab, India

**Keywords:** palmar lipoma, soft tissue tumor, deep palmar space, hand mass, surgical excision, grip dysfunction

## Abstract

Lipomas are common benign tumors, but their occurrence in the deep palmar space is rare due to anatomical constraints. We report the case of a 30-year-old male with a 2-year history of a progressively enlarging, painless swelling in the right palm, leading to impaired grip function. Imaging revealed a well-defined fatty lesion in the deep palmar space closely associated with the flexor tendons. The mass was excised completely with preservation of neurovascular and tendinous structures. Histopathology confirmed a lipoma. The patient recovered full hand function within four weeks. This case highlights the need to consider deep palmar lipomas in the differential diagnosis of chronic hand swellings and demonstrates that appropriate imaging and meticulous surgical excision can lead to excellent outcomes.

## Introduction

Lipomas are common benign mesenchymal tumors composed of mature adipocytes in lobules separated by fibrovascular septa [1]. Though they can arise in various sites, including subcutaneous and intramuscular compartments, only 1%–3.8% occur in the hand, making this location relatively rare [2, 3]. Given the hand’s compact anatomy, tumor expansion is restricted, yet lipomas may develop in both superficial and deep spaces like the carpal tunnel, Guyon’s canal, or deep palmar region [3, 4]. While usually asymptomatic, hand lipomas may cause compressive or mechanical symptoms depending on their size and location [4, 5]. We report a rare case of a giant lipoma in the deep palmar space causing progressive mechanical dysfunction without neurovascular involvement. Clinical, radiologic, surgical, and histopathologic findings are presented with relevant literature review.

## Case report

A 30-year-old male laborer presented with a progressively enlarging, painless swelling in the right palm over 2 years. Initially peanut-sized, the mass expanded to occupy most of the palmar surface, causing significant grip weakness and impaired hand function.

**Figure 1:**
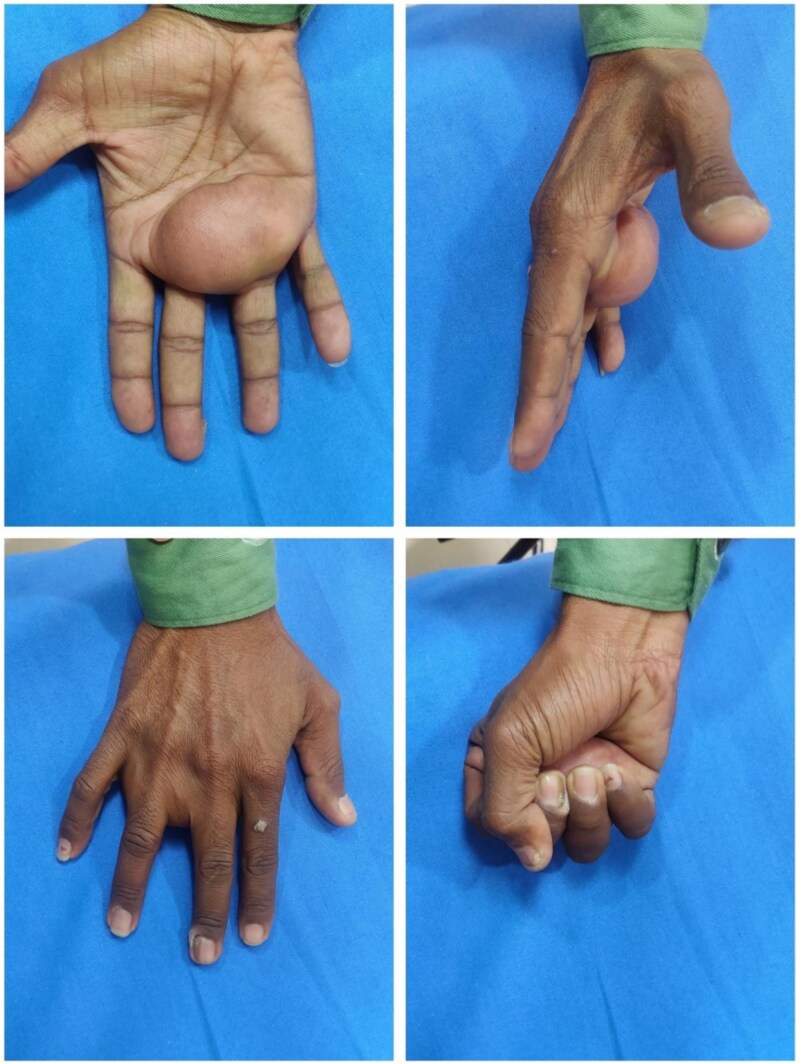
Preoperative clinical photographs showing a large, dome-shaped swelling in the central palm (top left), palmar projection from the lateral view (top right), and mild extension over the dorsum (bottom left). Grip weakness is evident on fist closure (bottom right).

On examination, a firm, non-tender, non-compressible 7 × 6 cm swelling extended from the second to fourth web spaces distally to the thenar crease proximally, spanning the entire width of the palm. Neurovascular examination was normal (Fig. 1).

NCCT imaging revealed a well-defined fatty lesion in the deep palmar space, adjacent to the flexor tendons. Surgical excision was planned based on clinical and radiologic correlation.

**Figure 2:**
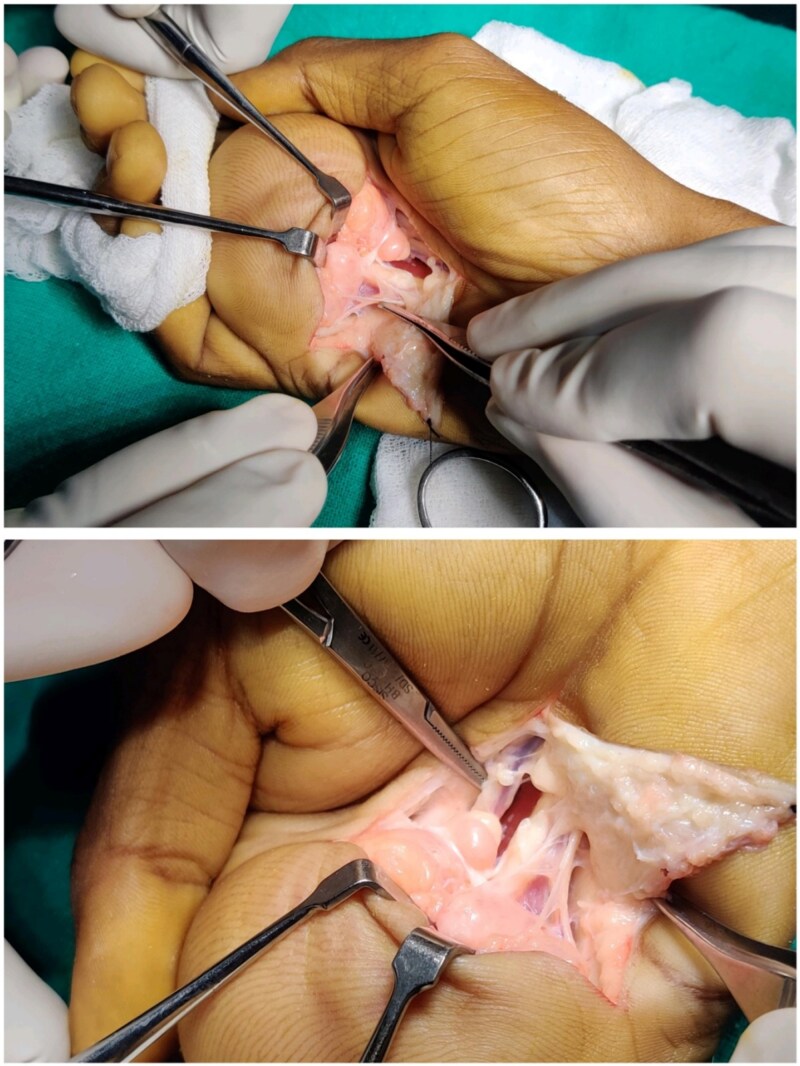
Intraoperative views showing deep dissection within the palmar space. The lipoma is identified closely adherent to flexor tendons and neurovascular structures, requiring meticulous dissection for en bloc removal.

**Figure 3:**
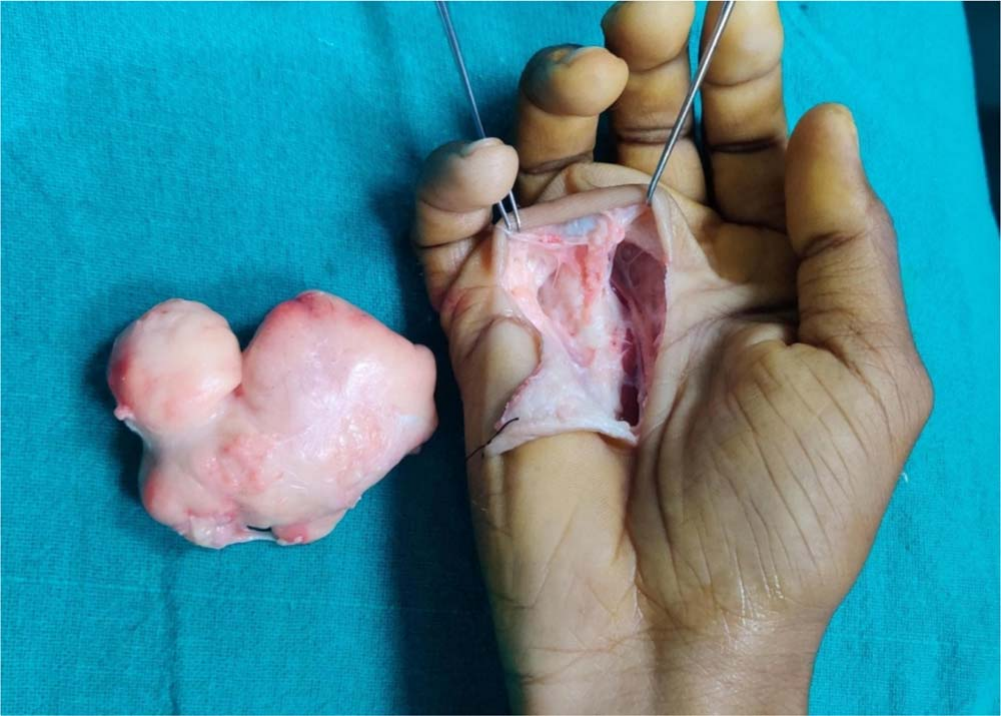
Gross specimen and post-excision cavity: A multilobulated, well-encapsulated yellow lipomatous mass measuring approximately 7 × 6 × 4 cm, shown next to the exposed deep palmar space after excision.

Surgical removal was done under brachial plexus block and tourniquet control. A curvilinear incision was made along the palmar and thenar creases. Dissection revealed a well-encapsulated, lobulated, yellow mass within the deep palmar space, extending dorsally toward the metacarpals and closely abutting flexor tendons and neurovascular bundles. The mass was carefully excised without damaging surrounding structures. It measured 7 × 6 × 4 cm (Figs 2 and 3).

The wound was closed in layers and dressed. Recovery was uneventful. Sutures were removed on postoperative day 10, and the patient was advised physiotherapy and scar massage.

**Figure 4:**
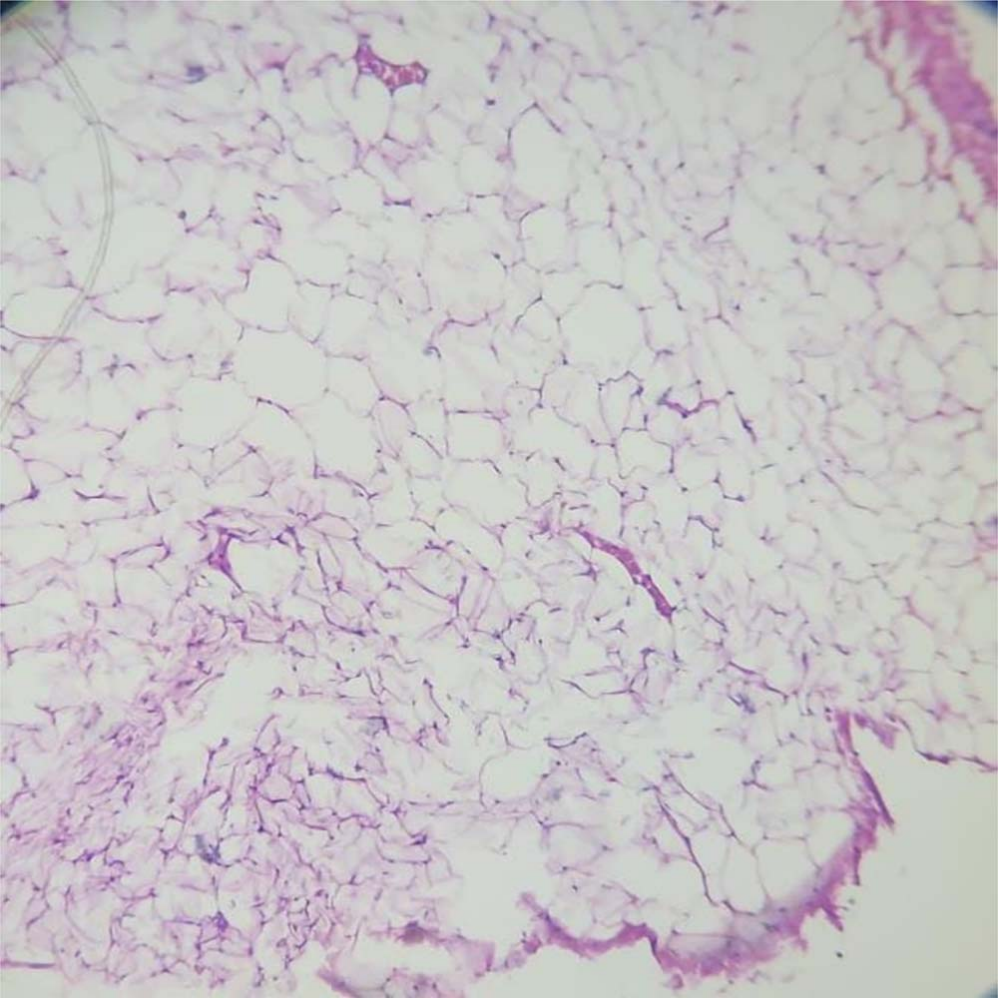
Histopathological section showing mature adipocytes arranged in lobules separated by thin fibrovascular septa, consistent with lipoma (H&E, 10×).

Mass was received in histopathology department. Grossly, the mass was soft, yellow, and encapsulated. Microscopy revealed mature adipocytes in lobules separated by thin fibrovascular septa, without atypia or lipoblasts, confirming diagnosis of lipoma (Fig. 4).

**Figure 5:**
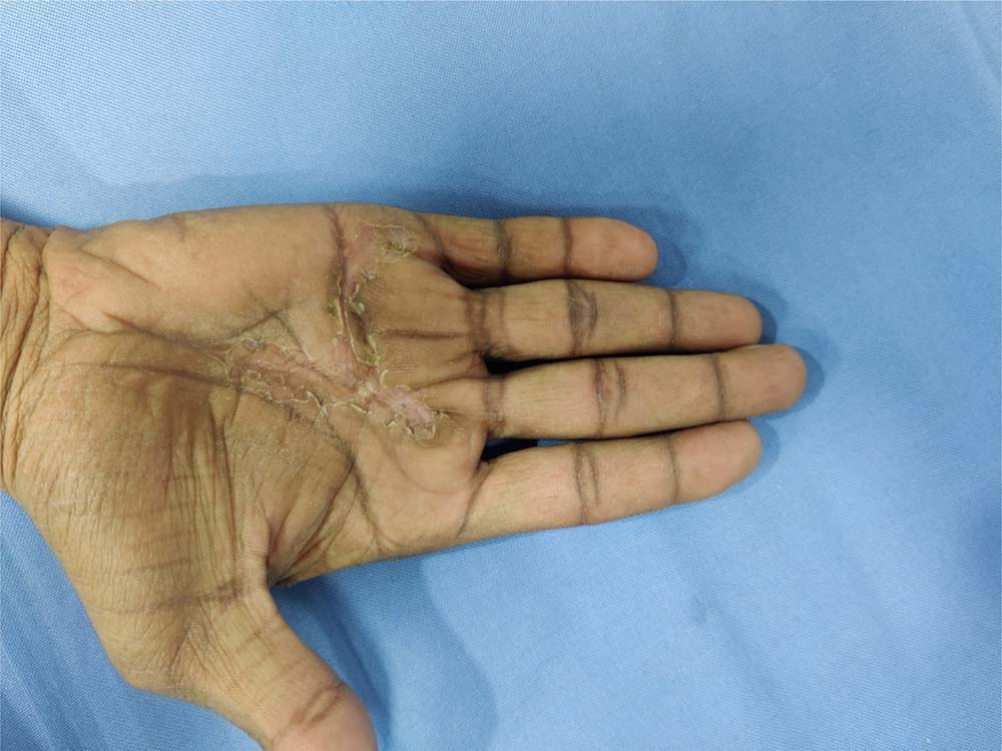
Postoperative clinical photograph at follow-up showing a well-healed surgical scar in the palmar region with complete restoration of hand contour and no residual swelling or deformity.

At 4 weeks, the patient had regained full grip strength and range of motion (Fig. 5).

## Discussion

Lipomas are benign tumors of adipose tissue and account for nearly half of all soft tissue tumors [1]. Although common elsewhere in the body, they are relatively rare in the hand, comprising only 1%–3.8% of benign hand tumors [2, 3]. The hand’s compact anatomy limits the space for tumor growth, making even small enlarging lesions clinically significant.

Lipomas in the hand can be superficial or deep-seated. While subcutaneous lipomas are more frequent, those arising in deep compartments such as the palmar space, carpal tunnel, or Guyon’s canal are uncommon and may resemble more aggressive pathologies like liposarcoma or vascular malformations [4, 5]. The deep palmar space is a rigid compartment that can silently accommodate large lesions with minimal external swelling, delaying diagnosis until functional symptoms emerge.

In this case, we report a giant lipoma (>6 cm) originating from the deep palmar space. Lesions >5 cm qualify as “giant” lipomas [6]. Despite their benign nature, such deep or enlarging masses warrant thorough evaluation to rule out malignancy. Our patient presented with mechanical grip impairment without classical neurovascular symptoms like numbness or tingling, commonly seen in carpal tunnel or Guyon’s canal involvement [4, 7]. This pattern of isolated mechanical dysfunction aligns with previous reports of deep hand lipomas compressing tendons while sparing neurovascular structures [3, 6].

Radiological imaging is key to diagnosis. While ultrasound is often the first-line tool due to its accessibility and cost-effectiveness, it is limited in assessing deep or complex lesions [7]. MRI is the preferred modality for characterizing soft tissue masses in the hand, given its superior soft-tissue contrast and multiplanar capability. Lipomas typically appear as well-circumscribed, homogeneously hyperintense masses on T1-weighted images, with signal suppression on fat-saturated sequences, distinguishing them from liposarcomas that may show septations, enhancement, or nodularity [1, 8]. Although MRI was initially performed in our case, it was unavailable for review; however, non-contrast CT revealed a well-defined, low-attenuation fatty lesion consistent with a lipoma, later confirmed on histopathology.

Surgical excision remains the definitive treatment for symptomatic or enlarging lipomas and serves both diagnostic and therapeutic purposes. In the hand, particularly in deep compartments, meticulous dissection is essential to preserve neurovascular and tendon structures [2, 3, 9]. In this case, en bloc removal of a 7 × 6 × 4 cm encapsulated mass was achieved without injury to the digital neurovascular bundles of the index, middle, and ring fingers. The lesion extended from the superficial palmar arch proximally to the distal web spaces, illustrating the capacity for silent growth within confined anatomical spaces.

Histologically, lipomas are composed of mature adipocytes arranged in lobules separated by delicate fibrovascular septa, without atypia, mitotic figures, or necrosis [4]. These features were observed in our specimen, ruling out malignancy. Although rare, malignant transformation into liposarcoma has been documented, particularly in large, deep, or recurrent tumors. Paarlberg *et al*. emphasized that soft tissue tumors exceeding 5 cm should always undergo histological examination to exclude malignancy [10]. In our case, the absence of clinical, radiologic, and histologic features of atypia confirmed a benign lipoma.

This case offers key insights for clinicians: deep palmar lipomas, though rare, should be considered in the differential diagnosis of chronic, painless hand swellings with functional deficits. While MRI remains the imaging modality of choice, CT in conjunction with clinical and intraoperative findings can often suffice for diagnosis and surgical planning. Complete surgical excision, with preservation of critical structures, typically results in full functional recovery. Moreover, all soft tissue tumors >5 cm—regardless of clinical presentation—should be histologically evaluated to rule out malignancy.

Given its deep location, large size, and silent progression, this case contributes valuable knowledge to the limited literature on deep palmar space lipomas.

In conclusion, giant lipomas of the deep palmar space are rare but may present silently with functional impairment rather than pain or neurovascular signs. While MRI is preferred, diagnosis can be made through clinical assessment, CT, intraoperative findings, and histopathology. Surgical excision is both diagnostic and therapeutic, emphasizing the need to consider deep lipomas in chronic, painless hand swellings and to plan surgery carefully to preserve function.

## Generative artificial intelligence (AI)

Artificial intelligence tools (such as ChatGPT) were used to assist with grammar, language refinement, reference formatting, and structural editing. No AI tools were involved in case analysis, data interpretation, clinical decision-making, or image generation. The case content and all images are original, and the authors take full responsibility for the scientific integrity of the manuscript.
